# Low bone density and post-operative outcomes following revision total shoulder arthroplasty

**DOI:** 10.1016/j.jsea.2026.100069

**Published:** 2026-08-03

**Authors:** Akin A. Adio, Muhammad Hamza Ilyas, Charbel Issa, Henry Gass, Andrew Horn, Hafiz F. Kassam

**Affiliations:** a Perelman School of Medicine at the University of Pennsylvania, Philadelphia, PA, USA; b Massachusetts General Hospital, Boston, MA, USA; c Lebanese American University Gilbert and Rose-Mary Chagoury School of Medicine, Beirut, Beirut Governorate, Lebanon; d Hoag Orthopedics, Newport Beach, CA, USA; e Hoag Orthopedic Institute, Newport Beach, CA, USA

**Keywords:** Osteoporosis, Osteopenia, Revision, Total shoulder arthroplasty, Low bone density, Joint replacement, Periprosthetic fracture, Loosening

## Abstract

**Background:**

Low bone density (LBD) may increase complication risk following revision total shoulder arthroplasty (rTSA), but the magnitude and time course of this risk in the revision setting have not been well characterized, with few studies reporting short-term or midterm outcomes. This study compared 90-day and 2-year outcomes after rTSA in patients with and without a documented diagnosis of LBD.

**Methods:**

We performed a retrospective cohort study including patients from January 1, 2005, through February 1, 2024. LBD was defined by a documented diagnosis of osteoporosis or osteopenia (International Classification of Diseases, Tenth Revision M80, M81, or M85.8) present at the time of the index revision. Adult patients undergoing rTSA with a diagnosis of LBD were matched to patients without such a diagnosis on demographics, comorbidities, and revision indication, so that documented LBD was the only variable differing between cohorts. Ninety-day post-operative outcomes and 2-year surgical outcomes were evaluated. Sub-group analyses assessed outcomes separately for osteoporosis and osteopenia cohorts. Relative risks with 95% confidence intervals were calculated.

**Results:**

After matching, 1,495 patients per cohort were included. At 90 days, a diagnosis of LBD was associated with higher rates of periprosthetic fracture (7.2% vs. 5.2%; relative risk (RR) 1.37, *P* = .028) and hospital readmission (24.6% vs. 17.9%; RR 1.38, *P* < .001). At 2 years, periprosthetic fracture remained significantly higher in the LBD cohort (11.2% vs. 8.7%; RR 1.29, *P* = .021), while mechanical loosening, dislocation, periprosthetic joint infection, and re-revision were not significantly different. In the osteoporosis sub-group (n = 963 per cohort), osteoporosis was associated with increased 2-year periprosthetic fracture risk (12.5% vs. 9.0%; RR 1.38, *P* = .015). The osteopenia sub-group (n = 640 per cohort) did not demonstrate a significant difference (9.8% vs. 8.9%; RR 1.11, *P* = .565).

**Conclusion:**

A documented diagnosis of LBD is associated with higher rates of periprosthetic fracture following rTSA. Sub-group analysis suggests that this association is primarily driven by patients with osteoporosis, whereas osteopenia alone was not associated with increased fracture risk. These findings highlight the importance of bone health characterization when evaluating risk profiles in patients undergoing rTSA.

Shoulder arthroplasty is increasingly indicated as both a primary and salvage treatment for fractures, avascular necrosis, glenohumeral arthritis, and rotator cuff pathology, with more than 100,000 shoulder arthroplasty procedures performed annually in the United States.^1^^,^^14^ Revision shoulder arthroplasty is indicated following failure of the index procedure due to complications such as component loosening, infection, glenoid wear, periprosthetic fracture, rotator cuff failure, and instability.^3^ As total shoulder arthroplasty continues to increase, the lifetime risk of revision has been reported to be as high as 25%.^14^ Importantly, revision shoulder arthroplasty is associated with inferior outcomes compared with primary procedures. Overall complication rates of up to 32% following revision surgery have been found, compared to 14% after primary total shoulder arthroplasty.^3^^,^^22^

Low bone density (LBD) is a skeletal disorder characterized by reduced bone strength and an increased risk of fracture.^6^ This encompasses both osteoporosis and osteopenia, conditions defined by decreased bone mineral density and increased bone porosity. Osteopenia represents bone density (BD) below normal reference values but not severe enough to meet diagnostic criteria for osteoporosis.^21^^,^^23^ Rates of LBD prior to total shoulder arthroplasty have been reported as high as 43.6%.^5^^,^^12^ Prior studies have demonstrated that patients with osteoporosis undergoing shoulder arthroplasty are at increased risk for both medical and implant-related complications, including periprosthetic fracture.^9^ However, limited evidence studies the short-term and midterm risks associated with LBD following revision total shoulder arthroplasty (rTSA). In primary shoulder arthroplasty, LBD has similarly been linked to the complications that define the end points of this study, including periprosthetic fracture and humeral and glenoid component loosening.^5^^,^^8^

This study aims to evaluate the association between LBD and post-operative outcomes following rTSA. This study compares 90-day post-operative complications and 2-year surgical outcomes between patients undergoing rTSA with and without LBD. We hypothesized that patients with a documented diagnosis of LBD would experience higher rates of complications, particularly periprosthetic fracture and hospital readmission, compared with patients without LBD.

## Materials and methods

This was a retrospective matched cohort study using the TriNetX Research Network, a federated database containing de-identified electronic health records from approximately 170 health-care organizations across the United States. The platform aggregates clinical information from hospitals and outpatient systems and allows large scale analysis of longitudinal patient outcomes. Diagnoses and procedures were identified using International Classification of Diseases, Tenth Revision codes and procedural coding systems. All data within TriNetX are fully de-identified and compliant with the Health Insurance Portability and Accountability Act.

### Cohort identification

Adult patients undergoing rTSA between January 1, 2005, and February 1, 2024 were identified. Revision procedures and revision indications were identified using standardized procedure and diagnosis codes (Supplemental Table S1). LBD was defined by the presence of International Classification of Diseases, Tenth Revision codes for osteoporosis (M80 and M81) or osteopenia (M85.8) prior to the index rTSA. Only diagnoses documented on or before the date of the index revision were counted; patients whose osteoporosis or osteopenia was first diagnosed after the revision were not classified as LBD and were excluded from the control cohort to reduce misclassification. The primary analysis compared patients with LBD to patients without either diagnosis. Secondary analyses separately evaluated osteoporosis only and osteopenia only cohorts compared with matched controls.

### One to one propensity-matched cohort

One to one propensity score matching was performed using logistic regression with nearest neighbor matching and a caliper of 0.01. Covariate balance was confirmed using standardized differences ≤0.01. Variables included age, sex, race, body mass index, obesity, diabetes mellitus, chronic kidney disease, liver disease, hyperlipidemia, nicotine dependence, vitamin D deficiency, revision indication, and pre-operative calcium and albumin levels. Antiosteoporotic medication use was not included as a matching covariate because such therapy was almost exclusively documented in patients with a LBD diagnosis and was therefore collinear with cohort assignment.

### Outcomes

The primary outcomes included ninety-day post-operative complications and two-year surgical outcomes following rTSA. Ninety-day outcomes included periprosthetic fracture, hospital readmission, venous thromboembolism, infection, acute kidney injury, wound dehiscence, transfusion, pneumonia, and urinary tract infection. Two-year outcomes included periprosthetic fracture, mechanical loosening, dislocation, periprosthetic joint infection, and re-revision arthroplasty. Each outcome was identified using its corresponding diagnosis and procedure codes (Supplemental Table S1).

### Statistical analysis

Relative risks with 95% confidence intervals and *P* values were calculated using the TriNetX analytic platform. Categorical variables were analyzed using chi square testing and continuous variables using Student t tests. Statistical significance was defined as *P* < .05. Because the study used only de-identified data, institutional review board approval was not required. Exact *P* values are reported for all comparisons, and secondary outcomes were interpreted in light of the number of comparisons performed.

## Results

A total of 6,595 adult patients undergoing rTSA were identified, of whom 1,668 had a documented diagnosis of LBD. After 1:1 propensity score matching, 1,495 patients per cohort were included. Following matching, demographic characteristics, medical comorbidities, and revision indications were well balanced between cohorts (all *P* > .05), as shown in Table I.

**Table I:** Characteristics of patients with and without low bone density.

Characteristics	Cohort	After matching		
		Patients	% patients	*P* value
Age at index	LBD Normal BD	1,495 (68.9 ± 9.83) 1,495 (69.1 ± 9.92)	100% 100%	.547
White	LBD Normal BD	1,336 1,327	88.771% 88.173%	.608
Not Hispanic or Latino	LBD Normal BD	1,247 1,218	82.857% 80.93%	.170
Black or African American	LBD Normal BD	101 107	6.711% 7.11%	.666
Hyperlipidemia, unspecified	LBD Normal BD	841 837	55.88% 55.615%	.883
Diabetes mellitus	LBD Normal BD	420 419	27.907% 27.841%	.968
Vitamin D deficiency	LBD Normal BD	416 418	27.641% 27.774%	.935
Chronic ischemic heart disease	LBD Normal BD	398 388	26.445% 25.781%	.678
Other chronic obstructive pulmonary disease	LBD Normal BD	304 296	20.199% 19.668%	.715
Nicotine dependence	LBD Normal BD	283 285	18.804% 18.937%	.926
Chronic kidney disease (CKD)	LBD Normal BD	283 302	18.804% 20.066%	.382
Diseases of liver	LBD Normal BD	232 231	15.415% 15.349%	.960
Dislocation of internal joint prosthesis	LBD Normal BD	328 330	21.794% 21.927%	.930
Infection and inflammatory reaction due to internal joint prosthesis	LBD Normal BD	272 273	18.073% 18.14%	.962
Periprosthetic fracture around internal prosthetic shoulder joint	LBD Normal BD	134 139	8.904% 9.236%	.751
Mechanical loosening of internal prosthetic joint	LBD Normal BD	267 268	17.741% 17.807%	.962
Calcium in serum, plasma, or blood	LBD Normal BD	1,416 (9.32 ± 0.593) 1,418 (9.31 ± 0.571)	94.086% 94.219%	.900
BMI	LBD Normal BD	1,305 (30.1 ± 7.18) 1,331 (30.2 ± 6.79)	86.711% 88.439%	.749
Albumin [mass/volume] in serum, plasma, or blood	LBD Normal BD	1,224 (3.97 ± 0.521) 1,237 (4 ± 0.502)	81.329% 82.193%	.087

*LBD*, low bone density; *BMI*, body mass index; *BD*, bone density.

### Primary analysis

At 90 days, patients with a diagnosis of LBD experienced significantly higher rates of hospital readmission than patients without LBD (24.6% vs. 17.9%; relative risk [RR] 1.38; *P* < .001). Periprosthetic fracture was also more frequent in the LBD cohort (7.2% vs. 5.2%; RR 1.37; *P* = .028). No significant differences were identified for venous thromboembolism (*P* = .116), infection (*P* = 1.000), acute kidney injury (*P* = .678), wound dehiscence (*P* = .121), transfusion (*P* = .062), pneumonia (*P* = .076), urinary tract infection (*P* = .257), or re-rTSA (*P* = .161). These results are summarized in Table II.

**Table II:** Ninety-day outcomes after revision total shoulder arthroplasty in patients with and without low bone density.

Low bone density vs. normal bone density (n = 1,495)					
Outcome	Incidence (%)		RR	95% CI	*P* value
	LBD	No LBD			
Venous thromboembolism	2.25% (34)	1.39% (21)	1.62	(0.81-3.21)	.116
Infection	2.74% (41)	2.74% (41)	1	(0.65-1.53)	1.000
Acute kidney injury	3.08% (46)	3.34% (50)	0.92	(0.62-1.36)	.678
Wound dehiscence	1.27% (18)	0.70% (≤10)	1.82	(0.81-4.10)	.121
Transfusion	1.81% (27)	1.00% (15)	1.8	(0.96-3.37)	.062
Pneumonia	1.67% (25)	0.94% (14)	1.79	(0.93–3.42)	.076
Urinary tract infection	3.01% (45)	2.34% (35)	1.29	(0.83–1.99)	.257
Readmission	24.62% (368)	17.86% (267)	1.38	(1.20-1.58)	**<.001**
Periprosthetic fracture	7.16% (107)	5.22% (78)	1.37	(1.03-1.82)	**.028**
Re-revision TSA	5.75% (86)	4.62% (69)	1.25	(0.92-1.70)	.161

*RR*, relative risk; *CI*, confidence interval; *TSA*, total shoulder arthroplasty; *LBD*, low bone density.

Bold indicates statistical significance.

At 2 years, patients with a diagnosis of LBD demonstrated a significantly higher incidence of periprosthetic fracture than those without LBD (11.2% vs. 8.7%; RR 1.29; *P* = .021). Rates of mechanical loosening (*P* = .606), dislocation (*P* = .282), periprosthetic joint infection (*P* = .276), and re-rTSA (*P* = .489) were not significantly different between cohorts. These findings are summarized in Table III.

**Table III:** Two-year outcomes after revision total shoulder arthroplasty in patients with and without low bone density.

Low bone density vs. normal bone density (n = 1,495)					
Outcome	Incidence (%)		RR	95% CI	*P* value
	LBD	No LBD			
Mechanical loosening	9.09% (136)	8.56% (128)	1.06	(0.84-1.34)	.606
Dislocation	15.65% (234)	14.25% (213)	1.1	(0.93-1.30)	.282
Periprosthetic fracture	11.24% (168)	8.69% (130)	1.29	(1.04-1.61)	**.021**
Periprosthetic joint infection	17.45% (261)	18.99% (284)	0.92	(0.79-1.07)	.276
Re-revision TSA	14.11% (211)	13.24% (198)	1.07	(0.89-1.28)	.489

*RR*, relative risk; *CI*, confidence interval; *TSA*, total shoulder arthroplasty; *LBD*, low bone density.

Bold indicates statistical significance.

### Two-year outcomes in the osteopenia sub-group

For each sub-group analysis, patients were separately matched 1:1 to their own controls without LBD. In a sub-group analysis limited to patients with a diagnosis of osteopenia matched to patients without LBD (n = 640 per cohort), no significant difference was identified in 2-year rates of periprosthetic fracture (9.8% vs. 8.9%; RR 1.11; *P* = .565). Mechanical loosening (*P* = .920), dislocation (*P* = .495), periprosthetic joint infection (*P* = .944), and re-rTSA (*P* = .745) were also similar between groups. These results are summarized in Table IV.

**Table IV:** Two-year outcomes after revision total shoulder arthroplasty in patients with osteopenia vs. normal bone density.

Low bone density vs. normal bone density (n = 640)					
Outcome	Incidence (%)		RR	95% CI	*P* value
	LBD	No LBD			
Mechanical loosening	8.44% (54)	8.59% (55)	0.98	(0.69-1.41)	.920
Dislocation	15.47% (99)	16.88% (108)	0.92	(0.71-1.17)	.495
Periprosthetic fracture	9.84% (63)	8.91% (57)	1.11	(0.77-1.58)	.565
Periprosthetic joint infection	19.69% (126)	19.84% (127)	0.99	(0.80-1.24)	.944
Re-revision TSA	13.44% (86)	14.06% (90)	0.96	(0.72-1.27)	.745

*RR*, relative risk; *CI*, confidence interval; *TSA*, total shoulder arthroplasty; *LBD*, low bone density.

### Two-year outcomes in the osteoporosis sub-group

Among patients with a diagnosis of osteoporosis matched to patients without LBD (n = 963 per cohort), osteoporosis was associated with a significantly higher 2-year risk of periprosthetic fracture (12.5% vs. 9.0%; RR 1.38; *P* = .015). No significant differences were identified for mechanical loosening (*P* = .128), dislocation (*P* = .705), periprosthetic joint infection (*P* = .054), or re-rTSA (*P* = .588). These findings are summarized in Table V.

**Table V:** Two-year outcomes after revision total shoulder arthroplasty in patients with osteoporosis vs. normal bone density.

Low bone density vs. normal bone density (n = 963)					
Outcome	Incidence (%)		RR	95% CI	*P* value
	LBD	No LBD			
Mechanical loosening	8.83% (85)	6.96% (67)	1.27	(0.93-1.73)	.128
Dislocation	15.16% (146)	15.78% (152)	0.96	(0.78-1.18)	.705
Periprosthetic fracture	12.46% (120)	9.03% (87)	1.38	(1.06-1.79)	**.015**
Periprosthetic joint infection	15.68% (151)	19.00% (183)	0.83	(0.68-1.00)	.054
Re-revision TSA	13.40% (129)	12.57% (121)	1.07	(0.85-1.34)	.588

*RR*, relative risk; *CI*, confidence interval; *TSA*, total shoulder arthroplasty; *LBD*, low bone density.

Bold indicates statistical significance.

## Discussion

This cohort study is, to our knowledge, the first large database analysis to evaluate the short-term and midterm impact of LBD on outcomes following rTSA. We found that a documented diagnosis of LBD was associated with significantly higher rates of early periprosthetic fracture and hospital readmission within 90 days of rTSA. At two years, a diagnosis of LBD was associated with elevated risk of periprosthetic fracture, while rates of mechanical loosening, dislocation, periprosthetic joint infection, and re-revision arthroplasty were not significantly different compared with patients with normal BD. Sub-group analyses demonstrated that this increased fracture risk was driven primarily by patients with osteoporosis, whereas patients with osteopenia exhibited similar midterm outcomes to those with normal BD. Collectively, these findings suggest that compromised bone quality, and more specifically osteoporosis, is an important determinant of fracture-related complications after revision shoulder arthroplasty.

Prior literature has demonstrated that osteoporosis is an important risk factor for periprosthetic fracture following primary shoulder arthroplasty. For example, Casp et al^5^ found significantly higher odds of periprosthetic fracture and revision within two years of both anatomic and reverse TSA compared with non-osteoporotic controls. These findings are consistent with growing lower-extremity arthroplasty literature.^2^^,^^17^ Given the cumulative effects of prior implants, bone loss, and stress shielding, the burden of compromised bone quality is expected to be even greater among patients undergoing revision shoulder arthroplasty.^7^^,^^19^ Notably, no significant increase in implant loosening was identified, consistent with Casp et al. This may reflect differences in fixation strategies used in patients with known osteoporosis.

The higher 90-day readmission rate associated with LBD likely reflects more than local bone quality. A diagnosis of osteoporosis frequently coexists with older age, sarcopenia, and multimorbidity, and may serve as a marker of overall frailty and diminished physiological reserve. Frailty is itself a strong, independent predictor of unplanned readmission after surgery, an association that is partly mediated by post-operative complications.^15^ Accordingly, surgeons evaluating patients for rTSA may treat a documented LBD diagnosis as a prompt for broader perioperative risk assessment, including frailty screening, medical comanagement, and structured care-transition planning, to help reduce readmission risk.

The association between osteoporosis and periprosthetic fracture is primarily attributable to compromised bone strength and altered microarchitecture in osteoporotic bone. Reduced bone mineral density and thinning of both cortical and cancellous bone diminish the ability of periprosthetic bone to tolerate bending and torsional loads.^10^ In the presence of an implant, stress is redistributed to the surrounding bone, increasing susceptibility to fracture even in the setting of low-energy trauma.^16^ Therefore, strategies aimed at improving bone quality may be useful in mitigating fracture risk. Antiosteoporotic therapy in patients with diagnosed osteoporosis has been associated with a reduced risk of all-cause revision and mechanical loosening following primary total shoulder arthroplasty.^11^ In the present study, antiosteoporotic medication use was almost entirely confined to the LBD cohort, and patients without a LBD diagnosis had essentially no documented antiosteoporotic therapy. This pattern also indicates that opportunities for pharmacologic bone health optimization were likely underused in this population.

In this cohort, patients with a diagnosis of osteopenia did not demonstrate an increased rate of periprosthetic fracture compared with those with normal BD. Although lower bone mineral density is a well-established risk factor for fragility fractures, the most substantial increases in fracture risk are seen at the diagnostic threshold for osteoporosis (T-score ≤ −2.5). Borowy et al^4^ found that there is marked escalation in fracture risk once a patient transitions from osteopenic to osteoporotic. Less dramatic increases in periprosthetic fracture risk are seen with milder reductions in BD. Additionally, Tomasevic et al^20^ showed that individuals with T-scores above the osteoporosis cut point have lower predicted fracture risk compared with those who meet criteria for osteoporosis. Together, these findings suggest that clinically meaningful periprosthetic fracture risk emerges primarily at the threshold of established osteoporosis rather than with osteopenia alone.

Because formal dual-energy X-ray absorptiometry is not always obtained before revision surgery, several practical surrogates can help surgeons screen for LBD. Opportunistic measurement of Hounsfield units on existing computed tomography imaging correlates with bone mineral density and can identify patients likely to have osteoporosis without additional cost or radiation, and computed tomography–based thresholds have demonstrated good diagnostic accuracy for osteopenia and osteoporosis in orthopedic populations.^8^^,^^13^ On plain radiographs, the cortical thickness of the proximal humeral diaphysis correlates with local bone mineral density and can help rule out osteoporosis and the deltoid tuberosity index offers a simple radiographic measure of proximal humeral bone quality.^17^^,^^18^ Incorporating these tools pre-operatively could make bone health assessment more actionable in the rTSA population.

This study has several limitations. First, its retrospective database design introduces the potential for selection bias, unmeasured confounding, and coding inconsistencies. BD status was determined from documented diagnosis codes rather than uniform, protocolized dual-energy X-ray absorptiometry. This limitation could attenuate observed associations, particularly among patients with milder reductions in BD. Additionally, we were unable to reliably distinguish between revision anatomic and reverse total shoulder arthroplasty. This may be clinically relevant given differences in underlying indications, implant biomechanics, and reported periprosthetic fracture risk between these procedures. Lastly, although this represents a large revision shoulder arthroplasty cohort, the overall sample size and event rates may limit statistical power to detect smaller differences between sub-groups.

## Conclusion

In this large cohort of rTSA, a diagnosis of osteoporosis was associated with increased periprosthetic fracture risk, whereas a diagnosis of osteopenia was not. These findings suggest that fracture risk in the revision setting is concentrated at the threshold of established osteoporosis and underscore the importance of pre-operative bone health assessment in this population. Pre-operative bone health optimization and proper implant selection and technique are paramount in avoidance of periprosthetic fracture in the revision shoulder arthroplasty population.

## Disclaimers:

Funding: No funding was disclosed by the authors.

Conflicts of interest: Hafiz F. Kassam is a consultant for Zimmer Biomet. Any additional authors, their immediate families, and any research foundations with which they are affiliated have not received any financial payments or other benefits from any commercial entity related to the subject of this article.
