# Supplementary material for: Low bone density and post-operative outcomes following revision total shoulder arthroplasty
Source: J Shoulder Elb Arthroplast. 2026 Aug 3;10(4):100069. doi: 10.1016/j.jsea.2026.100069 (PMC13545414; doi:10.1016/j.jsea.2026.100069)
Supplement: Supplementary file 1 [file mmc1.docx]

**Supplemental Materials**

**Low Bone Density and Postoperative Outcomes Following Revision Total Shoulder Arthroplasty**

**Supplemental Table S1.** Diagnosis and procedure codes used to define the study cohort, exposure, and outcomes in the TriNetX Research Network.

| **Category** | **Definition** | **Code(s)** |
| --- | --- | --- |
| Cohort — revision TSA | Revision of total shoulder arthroplasty | CPT 23473, 23474 |
| Exposure — osteoporosis | Osteoporosis | ICD-10 M80, M81 |
| Exposure — osteopenia | Osteopenia / low bone density, unspecified | ICD-10 M85.8 |
| Outcome — periprosthetic fracture | Periprosthetic fracture around internal prosthetic shoulder joint | ICD-10 M97.3-, T84.04- |
| Outcome — mechanical loosening | Mechanical loosening of internal prosthetic joint | ICD-10 T84.03- |
| Outcome — dislocation | Dislocation of internal joint prosthesis | ICD-10 T84.02- |
| Outcome — prosthetic joint infection | Infection/inflammatory reaction due to internal joint prosthesis | ICD-10 T84.5- |
| Outcome — venous thromboembolism | Deep vein thrombosis / pulmonary embolism | ICD-10 I82.-, I26.- |
| Outcome — surgical site infection | Infection following a procedure | ICD-10 T81.4- |
| Outcome — acute kidney injury | Acute kidney failure | ICD-10 N17.- |
| Outcome — wound dehiscence | Disruption of wound, not elsewhere classified | ICD-10 T81.3- |
| Outcome — transfusion | Transfusion of blood/blood products | CPT 36430 / ICD-10-PCS 30233N1 |
| Outcome — pneumonia | Pneumonia, unspecified organism | ICD-10 J18.- |
| Outcome — urinary tract infection | Urinary tract infection, site not specified | ICD-10 N39.0 |
| Outcome — re-revision | Subsequent revision total shoulder arthroplasty | CPT 23473, 23474 |

TSA, total shoulder arthroplasty; CPT, Current Procedural Terminology; ICD-10, International Classification of Diseases, Tenth Revision; PCS, Procedure Coding System. A trailing hyphen (-) indicates that all sub-codes within the category were included.
